# Abelacimab in Cancer-Associated Thrombosis: The Right Drug at the Right Time for the Right Purpose. A Comprehensive Review

**DOI:** 10.31083/j.rcm2410295

**Published:** 2023-10-19

**Authors:** Agnese Maria Fioretti, Tiziana Leopizzi, Daniele La Forgia, Raffaele De Luca, Donato Oreste, Riccardo Inchingolo, Pietro Scicchitano, Stefano Oliva

**Affiliations:** ^1^Cardio-Oncology Unit, IRCCS Istituto Tumori “Giovanni Paolo II”, 70124 Bari, Italy; ^2^Cardiology-Intensive Care Unit, Ospedale SS. Annunziata, 74121 Taranto, Italy; ^3^Diagnostic Radiology Unit, IRCCS Istituto Tumori “Giovanni Paolo II”, 70124 Bari, Italy; ^4^Surgical Oncology Unit, IRCCS Istituto Tumori “Giovanni Paolo II”, 70124 Bari, Italy; ^5^Interventional Radiology Unit, Ospedale Generale Regionale “F. Miulli”, Acquaviva delle Fonti, 70021 Bari, Italy; ^6^Cardiology-Intensive Care Unit, Ospedale della Murgia “Fabio Perinei”, Altamura, 70022 Bari, Italy

**Keywords:** cancer-associated thrombosis, factor XI inhibitors, abelacimab, bleeding risk

## Abstract

Cancer-associated thrombosis (CAT) is a devastating complication of cancer that can significantly impact a patient’s health and life. The incidence of CAT is approximately 20%, and 1 in 5 cancer patients will develop CAT annually. Indeed, CAT can promote pulmonary embolism and deep vein thrombosis, leading to increased morbidity and mortality that dramatically impact survival. CAT can also provoke delay or discontinuation of anticancer treatment, which may result in a lack of treatment efficacy and high costs for patients, institutions, and society. Current guidelines advocate direct oral anticoagulants (DOACs) as the first-line anticoagulant option in CAT. Compared to low-molecular-weight-heparins (LMWHs), DOACs are advantageous in that they typically have an oral route of administration, do not require laboratory monitoring, and have a more predictable anticoagulant effect. However, in patients with thrombocytopenia, renal failure, or those receiving anticancer regimens with potential for drug-drug interactions, LMWH is still the mainstay of care. The main limitation of current anticoagulant agents is related to bleeding risk (BR), both for DOACs and LMWHs. Specifically, DOACs have been associated with high BR in gastrointestinal and genitourinary cancers. In this challenging scenario, abelacimab, an anti-factor XI agent, could represent a viable option in the management of CAT due to its “hemostasis sparing” effect. The safe profile of abelacimab could be useful in patients with active malignancy and CAT, as long-term anticoagulant therapy is often required. Two ongoing international phase III trials (Aster and Magnolia) compare abelacimab with the standard of care (i.e., apixaban in patients with CAT and dalteparin in those with CAT and high BR, respectively). Abelacimab is a new and attractive anticoagulant for the management of CAT, especially in the insidious and critical scenario of active cancer patients with venous thromboembolism and high BR. The aim of this narrative review is to discuss the updated evidence on the performance of DOACs and LMWHs in the treatment of CAT and to focus on the potential role of abelacimab in CAT and its promising associated clinical trials.

## 1. Current Anticoagulant Treatments in Cancer-Associated Thrombosis

Venous thromboembolism (VTE), encompassing pulmonary embolism (PE) and deep vein thrombosis (DVT), is a frequent complication of cancer, leading to worsened survival [1]. It is estimated that the annual incidence of VTE in cancer patients is roughly 1 in 200 [2]. The risk of recurrence after a first episode of VTE and the bleeding risk (BR) in cancer patients during anticoagulant treatment is higher in comparison to those without cancer [3]. The occurrence of VTE in malignancy also contributes to increase healthcare expenses and worse quality of life for patients [4]. Difficulties in the treatment of VTE in cancer patients are in part attributable to the interplay between the tumor and the hemostatic system, in which the balance is often dramatically shifted toward a prothrombotic state [5]. Furthermore, a steady increase in the incidence of cancer-associated thrombosis (CAT) has been observed over the past 2 decades [6]. Low-molecular-weight heparins (LMWHs) and, more recently, direct oral anticoagulants (DOACs) are the main anticoagulant treatments used in the management of CAT. However, the BR related to the use of these drugs is not trivial and is exacerbated by the need for long-term anticoagulation [7]. Anticoagulant therapy lasting longer than 3 months is associated with an over 50% reduction in the recurrence of VTE (RVTE) risk in cancer patients [8]. The site of cancer plays a pivotal role in the efficacy and safety of anticoagulant treatment; for example, an increased risk of gastrointestinal (GI) hemorrhage was observed in patients with GI tumors in the Hokusai VTE Cancer and Select D trials but not in the Caravaggio trial [9]. However, fear of bleeding contributes to underuse and underdosing of eligible patients; moreover, high BR is the major contraindication to long-term anticoagulation. Accordingly, in the challenging setting of CAT, an emerging anticoagulant strategy able to mitigate BR would represent a superior treatment approach in CAT patients in whom BR is also higher compared to non-cancer patients as the risk of thrombosis. Indeed, a new generation of anticoagulants, the factor XI inhibitors (FXI), have a more favorable safety profile and may be what is warranted to erode physician and patient resistance to changing the standard of care [10]. Abelacimab, a monoclonal antibody that binds FXI and locks it in the inactive precursor conformation, is arising as an emerging anticoagulant option. This drug uncouples the thrombotic and hemostatic pathways, resulting in a safer anticoagulant profile that could be particularly useful in patients with CAT [11].

The purposes of this narrative review are to provide a comprehensive overview of the literature pertaining to the role of anticoagulants in CAT, to outline the expectations on the impact of abelacimab in CAT, and to suggest the possible indications for the use of abelacimab in clinical practice.

### 1.1 Low-Molecular-Weight Heparins

In the Canthanox study, 146 patients with cancer (53% with metastases) were randomized to receive warfarin or enoxaparin for secondary prevention. At 3-month follow-up, 21.1% of patients assigned to receive warfarin and 10.5% to enoxaparin experienced 1 major RVTE (*p* = 0.09), while 6 patients died due to hemorrhage in the warfarin group compared with none in the enoxaparin group (*p* = 0.07). Therefore, the results showed that enoxaparin was more effective and safer than warfarin in CAT patients [12].

To confirm the promising data of the Canthanox study [12], Lee *et al*. [13] published the Clot study, a benchmark trial in CAT. They enrolled 676 patients (67% with advanced disease) with CAT and assigned patients to receive dalteparin or warfarin. The probability of RVTE at 6 months was 17% in the warfarin arm compared to 9% in the dalteparin arm (*p* = 0.002), without significant difference detected in the rate of major bleeding (MB) (6% and 4% in warfarin and dalteparin group, respectively; *p* = 0.27) or any bleeding (14% and 19%, respectively, *p* = 0.09). The Clot study was the only trial that demonstrated a significant reduction in symptomatic RVTE with LMWH as compared to vitamin K antagonist (VKA).

In the Lite trial, 200 cancer patients with VTE were randomized to receive tinzaparin or usual care (VKA) for 3 months. At 12 months, the usual care group had an excess of RVTE compared to the tinzaparin group (16% versus 7%; *p* = 0.44). Bleeding, largely minor, occurred in 27% of patients in tinzaparin treated arm and 24% in VKA treated arm (*p* = 0.001) [14].

Enoxaparin was feasible, well-tolerated, and effective in 122 cancer patients with VTE in the Oncenox study, which compared enoxaparin alone versus initial enoxaparin followed by warfarin for 180 days. There was no significant difference in major or minor bleeding events and no fatal or intracranial hemorrhage (ICH). The overall compliance rate was high (97%) throughout the study. The long-term subcutaneous administration of LMWH was generally well-tolerated and was not an obvious deterrent to study participation or completion [15].

The Catch study was the largest (900 patients) trial comparing LMWH (tinzaparin) with VKA in cancer patients with VTE and lasted 6 months. RVTE occurred in 7.2% and 10.5% of the tinzaparin and VKA arms, respectively (*p* = 0.07) [16]. There were no significant differences in MB (12 patients receiving tinzaparin and 11 receiving warfarin; *p* = 0.77). On the contrary, a reduction was observed in clinically relevant non-major bleeding (CRNMB) with tinzaparin (49 of 449 patients receiving tinzaparin versus 69 of 451 patients receiving warfarin; *p* = 0.004) [17].

In the Clot trial [13], dalteparin was given at a full therapeutic dose for the first month, followed by 75%–80% of the full therapeutic dose from month 2 to month 6. In contrast, in Canthanox [12], Oncenox [15], Lite [14], and Catch [16, 17], full therapeutic doses of LMWH were given throughout the study. Whether this latter approach would have resulted in a lower risk of RVTE or higher BR in comparison to the Clot trial is unknown. Accordingly, these trials have made LMWH the standard of care for approximately 20 years. Overall, the aforementioned landmark clinical trials (Canthanox [12], Oncenox [15], Clot [13], Lite [14], and Catch [16, 17]) showed that LMWHs were superior to VKAs in preventing RVTE in cancer patients and had similar or improved BR profiles (see Table 1).

**Table 1.** **Trials comparing low-molecular-weight heparins to vitamin K antagonists**.

Study	Recurrent VTE	Recurrent VTE	Major bleeding	Major bleeding
	LMWH n/N (%)	VKA n/N (%)	LMWH n/N (%)	VKA n/N (%)
Canthanox	NA	NA	5/71 (7)	12/75 (16)
Clot	27/336 (8)	53/336 (16)	19/338 (6)	12/75 (16)
Oncenox	2/29 (7), 2/32 (6)	3/30 (10)	2/31 (7), 4/36 (11)	1/34 (3)
	1 mg/Kg; 1.5 mg/Kg		1 mg/Kg, 1.5 mg/Kg	
Lite	7/100 (7)	16/100 (16)	7/100 (7)	7/100 (7)
Catch	31/449 (7)	45/451 (10)	12/449 (3)	11/451 (2.4)

VTE, venous thromboembolism; LMWH, low-molecular-weight heparins; VKA, vitamin K antagonist; NA, not available; n, relative number; N, total number.

Nevertheless, the latest trials comparing DOACs with LMWHs in CAT downsized the use of LMWHs, as DOACs resulted in improvement with respect to therapeutic goals in long-term follow-up of patients with CAT [18].

### 1.2 Direct Oral Anticoagulants

The anti-Xa DOAC agents offer the advantages of an oral route of administration, fixed-dose, and no need for laboratory monitoring, resulting in less discomfort for patients and reduced costs for healthcare systems. Nonetheless, caution is recommended due to the high BR reported in GI and genitourinary (GU) cancers [19]. Main trials comparing DOACs with LMWHs in cancer patients with VTE effectively showed non-inferior efficacy but a higher rate of bleeding, especially in patients with GI and GU cancers (see Table 2).

**Table 2.** **Main trials comparing direct oral anticoagulants with low-molecular-weight-heparins**.

	Hokusai VTE cancer		Select-D		Adam-VTE		Caravaggio	
	Dalteparin	Edoxaban	Dalteparin	Rivaroxaban	Dalteparin	Apixaban	Dalteparin	Apixaban
VTE recurrence (N)	11.3% (524)	7.9% (525)	11% (203)	4% (203)	6.3% (142)	7% (145)	7.9% (579)	5.6% (576)
	Hazard Ratio 95% CI		Hazard Ratio 95% CI		Hazard Ratio 95% CI		Hazard Ratio 95% CI	
	*p* 0.71 (0.48–1.06) *p* –0.09		*p* 0.43 (0.19–0.99) no *p*		*p* 0.099 (0.013–0.78) *p* –0.0281		*p* 0.63 (0.37–1.07) *p * < 0.001	
Major bleed (N)	4% (524)	6.9% (522)	4% (203)	6% (203)	1.4% (142)	0% (145)	4% (579)	3.8% (576)
	Hazard Ratio 95% CI		Hazard Ratio 95% CI		Hazard Ratio 95% CI		Hazard Ratio 95% CI	
	*p* 1.77 (1.03–3.04) *p* 0.04		*p* 1.83 (0.68–4.96) no *p*		*p* Non estimable, *p* 0.138		*p* 0.82 (0.40–1.69) *p* 0.60	
Clinical not relevant major bleed (N)	11.1% (524)	14.6% (522)	4% (203)	13% (203)	4.2% (142)	6.2% (145)	6% (579)	9% (576)
	Hazard Ratio 95% CI		Hazard Ratio 95% CI		Hazard Ratio 95% CI		Hazard Ratio 95% CI	
	*p* 1.38 (0.98–1.94) no *p*		*p* 3.76 (1.63–8.96) no *p*		*p* Not provided		*p* 1.42 (0.88–2.3) no *p*	
Major bleed and clinical not relevant major bleed (N)	13.9% (524)	18.6% (522)	6.4% (203)	17.7% (203)	6.3% (142)	6.2% (145)	9.7% (579)	12.2% (576)
	Hazard Ratio 95% CI		Hazard Ratio 95% CI		Hazard Ratio 95% CI		Hazard Ratio 95% CI	
	*p* 1.4 (1.03–1.89) no *p*		*p* Not provided		*p* Not provided *p* 0.8816		*p* 1.16 (1.77–1.75) no *p*	
Death from any cause (N)	36.6% (524)	39.5% (522)	27.6% (203)	23.6% (203)	11% (142)	16% (145)	24.6% (579)	23.4% (576)
	Hazard Ratio 95% CI		Hazard Ratio 95% CI		Hazard Ratio 95% CI		Hazard Ratio 95% CI	
	*p* 1.4 (1.03–1.89) no *p*		*p* Not provided		*p* Not provided *p* 0.3078		*p* 0.82 (0.62–1.09) no *p*	

VTE, venous thromboembolism; CI, confidence interval; N, number of events, for example number of major bleedings.

The Hokusai VTE Cancer trial, an open-label (O-L) non-inferiority trial, randomized 1050 patients with active cancer and VTE to either edoxaban or dalteparin for at least 6 months and up to 1 year. Approximately 90% of patients had a solid tumor, and 70% in each arm received cancer treatment in the 4 weeks prior to the trial start. Metastatic disease was present in 60% and 57% of patients in the dalteparin and the edoxaban arms, respectively [20]. Overall, this trial showed that edoxaban had similar efficacy in preventing RVTE when compared to dalteparin (*p* = 0.006), but increased the risk of MB (*p* = 0.04), particularly upper GI bleeding in patients with GI tumors (edoxaban 3.8%, dalteparin 1.1%). Although the proportion of patients with GI cancer at baseline was comparable in both treatment arms (edoxaban 22.2%, 19.1% dalteparin), the median number of days from randomization to an MB event was 61 in the edoxaban group and 91 in the dalteparin group. Kaplan-Meier curves promptly separated after enrollment, thus supporting a difference in BR between edoxaban and dalteparin [21].

Select D was an O-L trial that randomized 406 patients with active cancer and VTE to rivaroxaban or dalteparin for 6 months. More than 95% of patients had solid cancers, roughly 70% followed anticancer regimens, while 58% had advanced disease in either treatment arm. The trial revealed that rivaroxaban was more effective than dalteparin at preventing RVTE at the expense of increased bleeding [22]. Most MB events were in the GI tract, and two-thirds of these GI bleeds were attributable to rivaroxaban (66.7%). CRNMB was significantly higher in the rivaroxaban arm as compared to the dalteparin arm. Moreover, the safety monitoring committee noted a non-significant increase in MB in 19 patients with esophageal or gastroesophageal cancers. Hence, subsequent enrollment of patients with either of these cancers was halted [23].

Patients with active cancer and residual VTE (REVTE) or index PE were randomized to rivaroxaban or placebo in the Select D 12m trial to explore RVTE beyond 6 months, but this further randomization was prematurely interrupted because of low recruitment (only 92 of 136 patients were randomized). Accordingly, Select D 12m was underpowered to detect a statistically significant reduction in RVTE with extended anticoagulation (300 patients were originally planned). The absence of VTE and/or index PE defined a population at low risk of recurrence [24].

The Adam VTE was an O-L trial that randomized 300 patients with active cancer and VTE to receive apixaban or dalteparin for 6 months. About 74% of patients received concurrent cancer treatment, and approximately 66% had metastatic disease. The trial detected a lower RVTE rate in the apixaban arm as compared to the dalteparin arm (*p* = 0.0281), while the rates of bleeding in both arms were low (*p* = 0.138). However, this trial included a small number of patients with upper GI cancers (11) as compared to the Hokusai VTE Cancer (54) [20] and Select D (40) [22] trials. The low rates of RVTE, MB, and CRNMB could be attributable to a difference in study design, sample size, patient selection, randomization, or management [25].

The Caravaggio trial was the largest (1170 patients) O-L trial and examined the use of DOACs in patients with active malignancy with VTE by comparing apixaban to dalteparin for 6 months. It excluded patients with brain primary or secondary tumors and/or leukemia. Approximately 60% of patients had advanced disease, and 60% were in concurrent anticancer treatment in each arm. This trial confirmed the results of the Adam VTE trial [25]: apixaban was non-inferior to dalteparin with respect to RVTE (*p* < 0.001) with a similar rate of MB events (*p* = 0.60). The most frequent site of MB was in the GI system, like in the Hokusai VTE trial [20] and in the Select D trial [22], with similar rates between the 2 arms, while CRNMB (mostly GI and upper airway) was increased in the apixaban arm [26].

## 2. Current Anticoagulants and Bleeding Risk in Cancer-Associated Thrombosis

### 2.1 Low-Molecular-Weight Heparins and Bleeding Risk

The function of long-term anticoagulant thromboprophylaxis in neuro-oncology patients remains uncertain since ICH often occurs even in the absence of anticoagulation. Among metastatic brain tumors, those originating from renal cell carcinoma and melanoma are particularly challenging due to their potentially life-threatening behaviors. ICH is also common in primary brain cancer, such as glioma, with an incidence of 10% to 20%. Concurrently, the risk of VTE is particularly high, with an incidence of 20% to 30%. LMWHs may be safer in patients with CAT and brain metastases but are associated with a 3-fold increased risk of ICH in patients with primary brain tumors [27].

The Prodige trial tested dalteparin 5000 units vs. placebo in 512 patients with glioma. Drugs were administered for 6 months and started within 4 weeks of surgery. Patients receiving LMWH had a reduced incidence of VTE (9 vs. 13 patients in the dalteparin and placebo groups, respectively) but increased ICH events (3 major bleeds vs. no bleeds in the dalteparin and placebo groups, respectively) [28].

The risk of bleeding was found to be 3 times higher in a group of 133 patients with glioma and poor prognosis who were treated with enoxaparin for VTE, suggesting that caution is warranted when considering therapeutic anticoagulation with this brain cancer [29].

Patients with brain metastases present with high rates of ICH, up to 20%. The results from an international 2-center retrospective cohort study of 96 patients with brain lesions receiving anticoagulation indicated comparable safety between DOACs and LMWHs, with a 12-month cumulative incidence of major ICH of 5.1% and 11.1%, respectively [30].

In a retrospective cohort study of 172 patients with primary or metastatic brain cancer randomized to treatment with LMWHs or DOACs, there was no significant difference in the cumulative incidence of any ICH (0% in the DOAC group, 36.8% in the dalteparin group) [31]. Similar results were found in another single-center study of 125 patients with either primary or metastatic brain cancer [32]. This study also confirmed significantly fewer MB events in the DOAC group than in the LMWH group, with a trend toward fewer ICH events. Recent trials [20, 22] comparing the efficacy and safety of edoxaban or rivaroxaban with dalteparin in CAT included very few patients with brain tumors. In the Hokusai VTE Cancer study [20], 6.5% of brain cancer patients in the edoxaban arm showed major hemorrhage compared to 9.3% of those in the dalteparin arm; however, no details were provided with respect to the nature of the hemorrhage or intracranial source. In the Select D cohort [22], there were only 3 primary brain tumor patients with no reported hemorrhages and no reported number of patients with advanced cancer. Other trials completely excluded patients with brain malignancy (i.e., Caravaggio [26] in the therapeutic setting and Cassini in the primary prophylaxis setting [33]).

The challenging decision to resume anticoagulation after an ICH event is likely attributable to an ICH recurrence rate of 10%. One retrospective study observed that enoxaparin once daily was associated with higher rates of bleeding, RVTE, and death [34]. These findings support the use of twice-daily LMWH injection; however, this may result in more cumbersome management of CAT.

### 2.2 Direct Oral Anticoagulants and Bleeding Risk

Although recent evidence establishes advances in CAT management, the unresolved issue of BR remains as DOACs have replaced LMWHs as the first-line treatment [35]. Indeed, DOACs exert their activity within the GI tract immediately after ingestion. In patients with reduced intestinal absorptive surface due to luminal lesions (i.e., from tumors or ulcers), these drugs may increase the risk of GI bleeding. Particularly, DOACs are absorbed by different sites throughout the GI tract. Edoxaban is primarily absorbed by the proximal small intestine, rivaroxaban by both the stomach and proximal bowel, and apixaban throughout the GI tract, including remarkable (>50%) absorption in the distal small intestine and ascending colon [36].

In general population studies, there is an increased BR that still causes concern among physicians [37].

At least 3 meta-analyses have been published comparing DOACs and LMWHs in terms of efficacy and safety in patients with cancer and VTE; these demonstrated that DOACs are associated with a trend toward increased GI BR (risk ratio [RR] 1.91: 95% confidence interval [CI] 0.96–3.82) [38, 39, 40]. Both Hokusai VTE Cancer [20] and Select D [22] trials detected an increased risk of GI MB (mostly upper-GI bleeding) with DOACs (edoxaban 6.9%, rivaroxaban 6%) compared to dalteparin (4% in both studies), largely in GI tumors. On the contrary, the Caravaggio trial [26] did not show a substantial difference in bleeding rates between apixaban and dalteparin. The causes for these disparities are still unclear; however, they could be due to different agents used and differences in the study populations [41].

In Hokusai VTE Cancer [20] and Caravaggio [26] trials, the percentage of patients with GI tumors was similar (about 30%, upper-GI cancers). Although more details, such as bleeding events by cancer type (i.e., GI cancer vs. non-GI cancer, intact luminal cancer), are essential to gain a deeper knowledge with respect to the use of DOACs in high-risk patients [42]. In the Hokusai VTE Cancer trial [20], 305 patients with GI cancer were included, of whom 54 (17.71%) had esophageal or gastric cancer. Findings were consistent in patients with luminal GI cancers (i.e., esophageal, gastric, or colorectal cancer) [43]. Edoxaban was associated with increased MB risk compared to dalteparin (hazard ratio [HR]: 2; 95% CI 1.09–3.66), mainly in patients with GI cancers (HR 4; 95% CI 1.5–10.6). In patients with GI cancer, upper GI bleeding accounted for 76.2% (16/21) of MB in the edoxaban arm and 0% (0/5) in the dalteparin arm. Out of those 16 upper GI MB events in those with GI cancer receiving edoxaban, 12 (75%) occurred in patients with unresected tumors [44].

In the Caravaggio trial [26], MB occurred in 3.8% and 4% of patients in the apixaban and dalteparin arms, respectively, and MB occurred in 9 patients with GI cancer in each treatment group. The clinical presentation of MB was severe or fatal in 6 patients receiving apixaban and 5 patients receiving dalteparin, and the clinical course was classified as severe in 5 patients in the apixaban arm and 7 patients in the dalteparin arm. MB occurred mainly in patients with luminal and non-resected GI cancers [45].

In the Select D trial [22], most MB events were located in the GI tract, mainly in esophageal and gastroesophageal tumors. There was a 3-fold relative increase in CRNMB with rivaroxaban vs. dalteparin. The hemorrhages were not negligible and satisfied at least 1 of the following criteria: requiring medical intervention or unscheduled contact with a clinician, interruption or discontinuation of study drug, or discomfort or impairment of daily life affairs. As a consequence, patients with GI cancer were thereafter excluded from enrollment as a precautionary measure. Based on residual deep vein thrombosis (REDVT) or index PE, Select D trial [22] patients were randomly assigned to rivaroxaban or a placebo for a further 6 months in order to assess RVTE and bleeding. The second randomization closed prematurely due to low recruitment (only 92 patients of the planned 300 were randomized). Consequently, the Select D 12m study [24] results were not statistically significant, and the trial was underpowered. However, MB and CRNMB rates were 0% and 0% in the placebo arm and 5% and 4% in the rivaroxaban arm, respectively. Therefore, bleeding as a main complication of anticoagulation with DOACs should be taken into consideration even after the first 6 months of treatment, as bleeding events may still occur.

Houghton *et al*. [46] showed that in 1392 patients with VTE (35.8% with GI cancers), apixaban had a higher rate of MB in the luminal GI-cancer group as compared to that in the non-GI cancer group (15.59 vs. 3.26 per 100 person-years; *p* = 0.004) and a lower rate of CRNMB in comparison with rivaroxaban in patients with GI tumor (3.83 vs. 9.40 per 100 person-years; *p* = 0.03). Patients treated with rivaroxaban in the luminal GI cancer group had a MB rate similar to that of patients with non-GI cancers (2.04 vs. 4.91 per 100 person-years; *p* = 0.37).

GU bleeding events may be frequent in patients with CAT receiving DOAC treatment, especially with rivaroxaban. Indeed, in the Select D trial [22], hematuria resulted a CRNMB in 1 patient in the dalteparin arm and in 9 patients in the rivaroxaban arm. In the Hokusai VTE Cancer trial [20], 13.2% of enrolled patients had GU cancers, and GU bleeding events were too few for conclusive analysis [38]. One meta-analysis showed an increased risk of GU MB with DOACs compared to LMWHs (RR 4.99; 95% CI 1.08–23.08). The risk of CRNMB was also found to be increased (RR 2.2; 95% CI 1.33–3.63) [39]. The site of bleeding may correlate with the cancer site, but most studies (except for Hokusai VTE Cancer [20]) have not published subgroup analyses according to tumor type to validate this hypothesis. As such, a meta-analysis of DOAC trials based on each cancer subtype would be informative [40]. Grounded on the available evidence, DOAC use in CAT is associated with a high risk of GI bleeding, and the excess bleeding was limited to GI-cancers, luminal and non-resected. In this clinical panorama, LMWHs remain the treatment of choice, and DOACs should be used with caution. Likewise, considerations are reasonable for patients with high GU BR, such as those with active and non-resected GU lesions, recent GU cancer surgery, or recent (<6 months) GU MB. Although both DOACs and LMWHs are cleared in the urinary tract, biologically active DOACs are present in the urinary tract, while LMWHs (given the absence of antithrombin in the urine) would not exert any anticoagulant effect and would be expected to be safe [47]. Although DOACs offer the advantage of a longer action and easier use [48], the bleeding risk persists.

The Select D [22] and the Caravaggio [26] trials excluded patients taking any strong inducer or inhibitor of cytochrome P450 3A4 (CYP3A4) or P-glycoprotein (P-gp), and Hokusai VTE Cancer [20] excluded only patients using specific strong P-gp-inhibiting drugs with dose adjustment to edoxaban 30 mg once daily for patients taking other strong P-gp inhibitors. Accordingly, patients with potential increased BR were not assessed in these trials. Moreover, Caravaggio [26] excluded patients with leukemia and brain tumors, primary or metastatic, which are known for high BR [49].

According to the real-world data, there is no difference in MB risk between DOACs and LMWHs, although observational studies have many limitations, such as small sample sizes and selection bias. Moreover, decisions regarding the management of anticoagulant treatment in CAT were left to the discretion of attending physicians and patient preference [50].

## 3. Pharmacological Profile of Abelacimab

Abelacimab is a fully potent human monoclonal antibody of 51 serine-type plasma protease, highly selective, designed to bind the catalytic domain of FXI and lock it in its zymogen (inactive precursor) conformation by that means preventing its activation by FXIIa or thrombin (FIIa). It demonstrates dual inhibitory activity against factor XI and its activated form, factor XIa. Abelacimab can be administrated intravenously (IV) to fast-reach inhibition of FXI activity and then used subcutaneously (SC) monthly to maintain almost complete inhibition in a chronic setting [51]. It is still under investigation; therefore, any official approval for daily clinical application could be provided. It has a rapid onset and slow offset of action and neither requires renal clearance nor presents issues with hepatic metabolism. In addition, it poses no risk of drug-drug interactions. Indeed, the results of anthos therapeutics (ANT)-003 (healthy volunteers) and ANT-004 (patients with atrial fibrillation) demonstrated that IV and once monthly SC administration of abelacimab are safe and well-tolerated, producing marked and sustained FXI inhibition beyond 4 weeks [52].

Both the displayed favorable pharmacokinetic and pharmacodynamic properties and the monthly dosing demonstrate the feasibility of abelacimab in comparison to the existing oral anticoagulant agents, which require once or twice daily dosing, resulting in common peak-trough excursions in plasma concentrations, balancing efficacy with BR. Importantly, its easy use overcomes the pain and discomfort associated with the once or twice-daily injection, often observed with LMWHs.

Moreover, abelacimab was also tested in severely obese patients, resulting in a moderately lower duration of FXI inhibition. However, further evidence is needed to explore whether dose adjustment in patients with severe obesity is required [53].

Overall, the absence of a specific antidote, the slow offset of action, and the presence of comorbidities could increase BR in the use of abelacimab, mainly in cancer patients who require long-term anticoagulant treatment and need additional investigations. Strategies to manage life-threatening bleeding, not necessarily abelacimab-related, severe thrombocytopenia, emergent surgery, or interventional procedures, are still to be determined [54].

## 4. Evidence on the Use of Abelacimab in Cancer-Associated Thrombosis

### 4.1 The Theory Disproof of Separating Thrombosis from Hemostasis by FXI Inhibition

Thrombin acts as a central action in hemostatic and thrombotic pathways. There are 2 strategies to target this protease to treat or prevent thrombosis. It directly blocks protease active sites (DOACs) or indirectly blocks it by potentiating antithrombin-mediated protease inhibition (LMWHs). These strategies are effective but carry a risk of MB since they do not distinguish the thrombin that drives thrombosis from that required for hemostasis. Consequently, the use of such anticoagulants involves a striking and somewhat perilous balance between a significant antithrombotic effect and an acceptable anticoagulant one [55].

A novel class of drugs has been recently assessed in cancer patients with VTE and appeared of great interest: FXIis, which might achieve an antithrombotic effect with minimal or no compromise of hemostasis. The reluctance of physicians to provide optimal anticoagulant treatments due to the dreaded increased BR results in undertreatment. In addition, per the Garfield Registry, 30.4% of patients discontinued anticoagulation within 9 months from the index VTE event [56]. Furthermore, we should expect a marked increase in CAT diagnosis due to the longer life of cancer patients to the recent improvements in cancer treatments [57].

**Fig. 1.**
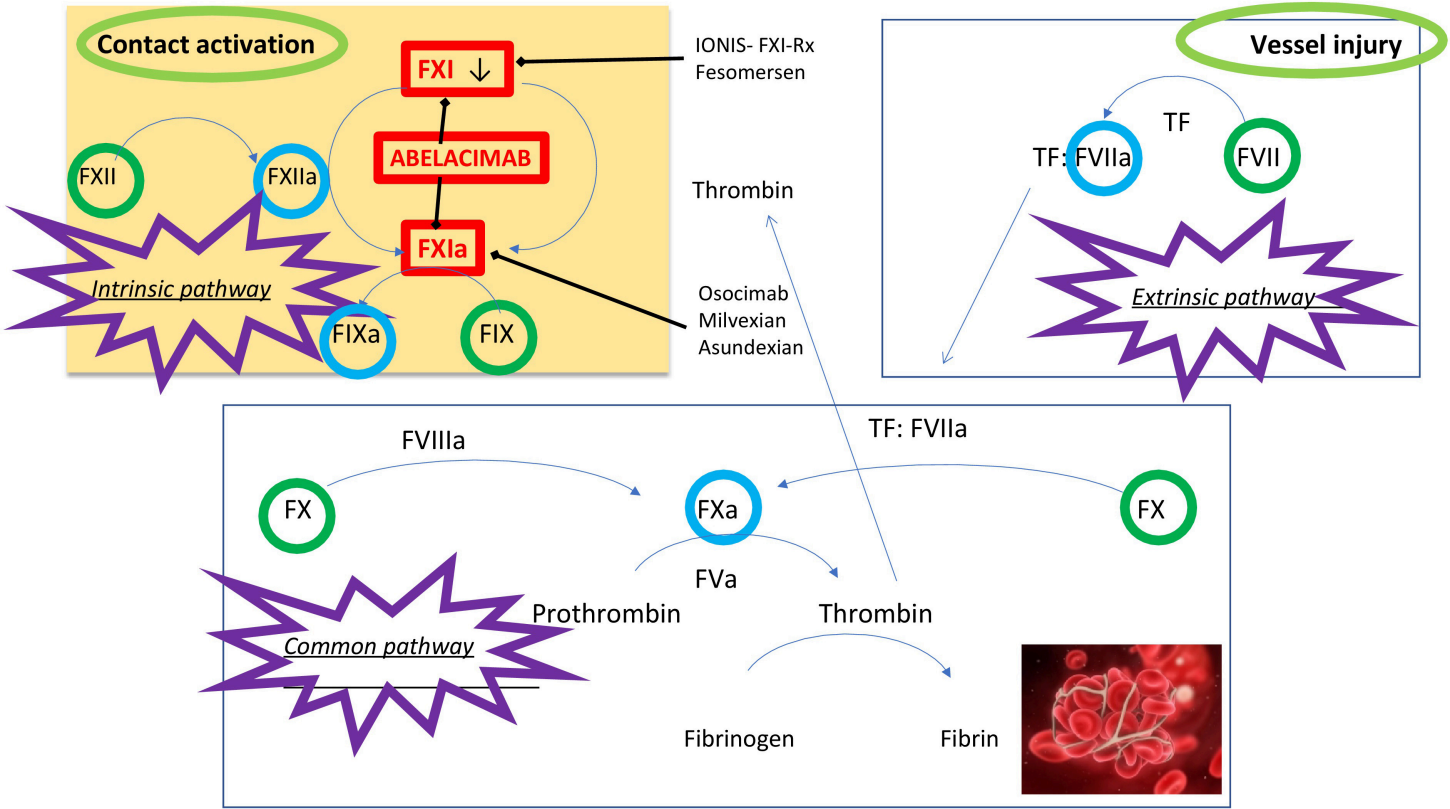
**Coagulation cascade and Factor XI inhibitors**. FXI(a), factor XI(a); FXII(a), factor XII(a); FIX(a), factor IX(a); FVII(a), factor VII(a); FVIIIa, factor VIIIa; FX(a), factor X(a); FVa, factor Va; TF, tissue factor.

The coagulation cascade starts with the activation of factor X (FX) by the complex tissue factor (TF)-factor VII (FVII) that converts prothrombin to the active thrombin, which, in turn, catalyzes the conversion of fibrinogen into fibrin. The indirect activation of FX occurs via the activation of factor IX (FIX) in the presence of its cofactor, factor VIII (FVIII), since FIX can activate FX forming an amplification loop. A second amplification route is made by the thrombin-mediated activation of FXI that activates FIX. In the presence of factor V (FV), activated FX converts prothrombin into thrombin which, in turn, catalyzes the conversion of fibrinogen into fibrin (see Fig. 1).

FXI is a component of the intrinsic pathway of coagulation and links it to the contact system (factor XII, prekallikrein, and high-molecular-weight kininogen) that is epidemiologically involved in thrombosis and may be less crucial for hemostasis [58]. High levels of FXI have been shown to increase the risk of DVT 2-fold. On the contrary, its deficiency, known as hemophilia C, unlike hemophilia A or B, seldom manifests as spontaneous bleeding. Bleeding in hemophilia C usually occurs only after trauma or surgery, and it typically causes only mild bleeding [59]. FXI-deficient patients consistently have prolonged activated partial thromboplastin time, with prolongation longer than that observed with deficiencies of FVIII or FIX. Epistaxis and menorrhagia are relatively frequent in patients with FXI deficiency: 59% of FXI-deficient women reported symptoms compared to only 10% in the general population [60].

Noteworthy, humans with severe FXI deficiency may experience excessive injury-induced bleeding, especially when trauma involves tissues that are rich in fibrinolytic activity, such as the nose, mouth, and GU tract. Indeed, hemorrhage at other sites, such as the central nervous system, GI tract, and muscles, is less frequent and tends only to occur in individuals where FXI levels are lower than 10% of normal and spontaneous bleeding is rare [61]. Thrombin generation assay is a clinical tool able to differentiate between FXI-deficient individuals with a history of bleeding (“bleeders”) and those without (“non-bleeders”) in order to predict the possible risk of bleeding in FXI-deficient individuals undergoing surgery [62].

A cohort of 10,193 patients tested for factor XI were enrolled from the database of Clalit Health Services, i.e., the largest health care provider in Israel. Patients were classified into 3 categories according to factor XI activity degree: normal, mild, and moderate-to-severe. Factor XI deficiency was associated with decreased incidence of cardiovascular events (CVEs) and VTE. Moreover, this study postulated a dose-response relationship between factor XI deficiency and a J-shaped relationship between factor XI deficiency and CVEs [63]. Therefore, mild-moderate factor XI deficiency seemed to promote the greatest cardiovascular benefits, while higher FXI activity is a risk factor for VTE. Among anticoagulant factors (IX-XIII), only elevated levels of FXI was independently associated with an increased risk of VTE development. This association was similar for idiopathic and secondary VTE among older and younger and was stronger for PE and DVT [64].

The Leiden Thrombophilia Study (LETS) demonstrated that 10% of the general population with the highest FXI plasma was shown to have an approximately 2-fold increase in the risk of VTE in comparison with the remaining 90% of the population. These observations suggested that the FXI amplification pathway is less essential for normal hemostasis *in vivo*, although FXI seems to play a main function in thrombosis processes [65].

Moreover, the Study of Myocardial Infarction Leiden (SMILE) outlined that elevated FXI levels in men younger than 70 years and with a first myocardial infarction (MI) event induced a 1.8-fold increase in the risk of MI [66]. In contrast, a small study that enrolled 96 patients with severe FXI deficiency documented that the observed incidence of MI was similar to the expected incidence in the general population, suggesting that severe factor XI deficiency confers no protection against thrombosis [67]. Furthermore, in 78 out of 100 patients under 55 years who had undergone evaluation for a hypercoagulable state, 22% showed FXI plasma levels higher than the 95th percentile, which was associated with an increased odds ratio of 5.3 [68].

Conversely, a remarkably reduced incidence of ischemic stroke, but not of MI, has been observed in 115 patients with FXI deficiency as compared to the general population, mirroring apparent protection against ischemic ictus cerebri due to reduced thrombin generation and augmented lysis of blood clots formed or embolized into cerebral arteries [69]. In addition, the use of FXI concentrates must be carefully assessed as higher BRs associated with older age and excess weight were observed [70].

A cohort of 219 patients with severe FXI deficiency showed a significantly reduced incidence of DVT. These findings could imply that severe FXI deficiency could also be protective from arterial and venous thrombosis [71]. Collectively, these observations favor the targeting of FXI for the development of efficient antithrombotic therapies with improved safety as compared to current anticoagulants that target FX, FII, or multiple factors and carry considerable bleeding risk.

FXI contributes to thrombin generation and promotes inhibition of fibrinolysis and can be regarded as a procoagulant, and as an antifibrinolytic component, thus its severe deficiency might predict hypocoagulability as well as enhanced fibrinolysis. Notably, FXIis uncouple physiologic hemostasis from pathologic thrombosis (see Table 3). In addition, FXI belongs to the coagulation cascade of mammals but not of other vertebrates as the result of ontogenic thread, leading to thrombin generation by 2 pathways: the contact system and the extrinsic pathway. The former, FXI-mediated, is triggered by the exposure of blood to extracorporeal surfaces, whereas the latter, TF-mediated, is due to vessel injury. Accordingly, FXI provides a consistent antithrombotic effect when thrombosis is not TF-related [56]. However, hemostasis *in vivo*, unlike *in vitro*, is principally directed by TF, following the extrinsic pathway. Abelacimab, if established, could improve the main unmet needs in CAT: its long half-life could allow the monthly dosage to favor the patient’s persistence, the parenteral administration could avoid the potential GI bleedings, its metabolism not primarily liver or kidney related could benefit cancer patients with severe kidney or liver failure and the contact system activation could be useful in indwelling line-related thrombosis [72].

**Table 3.** **Benefits and limitations of factor XI as a target**.

Benefits of factor XI as a target	Limits of factor XI as a target
Elevated XI levels are associated with an increased risk of VTE, stroke, and myocardial infarction	A high level of inhibition could increase bleeding with trauma or surgery, especially if the nose, mouth, or urinary tract is involved
Severe factor XI deficiency is associated with modest bleeding diathesis	A high level of inhibition could cause or aggravate bleedings at particular sites, such as gynecological
Factor XI inhibition could hamper the contact activation and the thrombin-mediated feedback activation on thrombosis	If thrombosis is TF-mediated antithrombotic effect could be modest

VTE, venous thromboembolism; TF, tissue factor.

### 4.2 Perspectives

Anticoagulants targeting FXI seem to play a non-essential role in physiologic hemostasis, even though they strongly contribute to pathological thrombosis, showing that the pathways enrolled in hemostasis and thrombosis are distinct. Introducing a paradigm shift in anticoagulant therapy would be a long-acting FXIi able to target maximal efficacy with minimal BRs [73].

Nevertheless, anti-FXIi drugs are still under investigation, and their use as a safe, effective, and easy-to-administer alternative anticoagulant option to DOACs and LMWHs is still to be proved. The generic versions of DOACs will be cost-effective, leading to increased utilization. Thus, anti-FXIi agents will need to be explored to determine their ideal indications and may be used in scenarios where DOACs are contraindicated, or their use is not well set [54].

Medications that inhibit FXI are at various stages of development and testing in humans; none has achieved phase 3 evaluation. New drugs directed against FXI include inhibitors of biosynthesis, antibodies, small molecules, and derivatives of naturally occurring inhibitors [74]. They present different advantages and disadvantages since they are endowed with different mechanisms of action and pharmacological properties (see Table 4).

**Table 4.** **Anticoagulants targeting FXI features**.

	Antibodies	Small molecules	Natural inhibitors	Antisense oligonucleotides	Aptamers
Mechanism	Bind target protein	Bind target protein	Bind target protein	Block biosynthesis	Bind target protein
Administration route	IV or SC	IV or oral	IV	SC	IV or SC
Administration frequency	Monthly	Daily	Daily	Weekly to monthly	Daily
Onset of action	Rapid (hours or days)	Rapid (minutes or hours)	Rapid (minutes)	Slow (weeks)	Rapid (minutes or hours)
Offset of action	Slow (weeks)	Rapid (minutes to hours)	Rapid (hours)	Slow (weeks)	Rapid (minutes to hours)
Renal excretion	No	Yes	Uncertain	No	No
Cytochrome P450 metabolism	No	Yes	Uncertain	No	No
Potential for drug-drug interactions	No	Yes	Unknown	No	No

FXI, factor XI; IV, intravenously; SC, subcutaneously.

### 4.3 Abelacimab in Cancer-Associated Thrombosis

A robust ongoing phase 3 clinical program includes 2 complementary trials (Aster, NCT05171049, and Magnolia, NCT05171075): they will enroll 1655 and 1020 patients from 220 sites in more than 20 countries, respectively. This is the largest scientific project investigating the use of anticoagulants in the management of CAT. The aim of both trials is to assess the use of abelacimab vs. standard of care (DOACs in patients with CAT and LMWHs in GI/GU cancer patients). The primary endpoint is time to the first event of centrally adjudicated RVTE, consisting of a new proximal DVT, new or fatal PE, including unexplained death for which PE cannot be ruled out. Secondary endpoints encompass time to the first event of the International Society on Thrombosis and Hemostasis-adjudicated major or CRNMB events and the net clinical benefit defined as survival without RVTE or major or CRNMB events [75].

Aster is an international multicenter, randomized, O-L blinded endpoint evaluation exploring the effects of abelacimab compared to apixaban on RVTE and bleeding in patients with a confirmed CAT other than basal cell carcinoma or squamous cell carcinoma of the skin in whom DOAC treatment is indicated. Abelacimab 150 mg will be administrated IV on day 1 and SC monthly thereafter for up to 6 months; apixaban 10 mg will be administrated per os, twice daily for the first 7 days, followed by 5 mg twice daily for up to 6 months [76].

Magnolia is an international multicenter, randomized, O-L, blinded endpoint study investigating the effects of abelacimab compared to dalteparin on RVTE and bleeding in patients with GI/GU cancer, in whom DOAC treatment is not recommended due to the high BR with non-resectable, locally or regionally invasive metastatic or non-metastatic GI/GU tumors, who will not undergo intended curative surgery during the study. Abelacimab will be administrated as in the Aster trial. Dalteparin will be given SC at 200 IU/kg/day for the first month and then at 150 IU/kg/day for up to 6 months. The patients enrolled are required to have confirmed symptomatic or incidental proximal lower extremities acute DVT and/or established symptomatic PE, an incidental PE in a segmental or larger pulmonary artery [77].

## 5. Conclusions

VTE is a common and fatal complication of cancer. DOACs and LMWHs are still the mainstays of treatment. However, their use leads to a substantial increase in bleeding that remains a deterrent to optimal anticoagulation for physicians. The landscape of cancer therapy and survival is rapidly changing; accordingly, anticoagulant therapy in CAT must keep pace since safer anticoagulation strategies are required. Current anticoagulants target factors of the common pathway of the coagulation cascade, while a more upstream inhibition could be a promising treatment approach with the aim of preventing thrombosis without impairing hemostasis.

To broaden the anticoagulant options available, research is now focused on inhibiting the intrinsic pathway, with FXI emerging as the most encouraging candidate target. Mounting evidence indicates that the therapeutic armamentarium will be widened by anti-XI agents such as abelacimab. Its “hemostasis sparing” profile could be particularly useful in the most feared issue of patients with CAT and high BR, uncoupling the desired effective antithrombotic effects from the deleterious anti-hemostatic ones.

CAT patients deserve this appealing and potentially safer anticoagulant solution since a long-term and, sometimes, indefinite treatment is required. Moreover, the once-monthly SC dosing of abelacimab could improve patient compliance, increase treatment satisfaction, and reduce patient discomfort. The new paradigm for the management of CAT may ultimately find abelacimab as its expected “holy grail”, still to be confirmed.
